# Granulomatosis with polyangiitis masquerading as lung cancer with meningeal metastasis: a case report and literature review

**DOI:** 10.3389/fmed.2026.1970702

**Published:** 2026-10-21

**Authors:** He Zhou, Xiaoyu Zhang, Dunya Yang, Yanyan Jiang, Yun Lin, Lin Ye, Xiujuan Yan, Jie Li, Qunxiang Chen, Xi Chen

**Affiliations:** 1 Department of Neurology, Fuzong Clinical Medical College of Fujian Medical University, Fuzhou, Fujian, China; 2 Department of Neurology, 900th Hospital of PLA Joint Logistic Support Force, Fuzhou, Fujian, China; 3 Department of Oncology, Fuzong Clinical Medical College of Fujian Medical University, Fuzhou, Fujian, China; 4 Department of Oncology, 900th Hospital of PLA Joint Logistic Support Force, Fuzhou, Fujian, China

**Keywords:** anti-neutrophil cytoplasmic antibody, differential diagnosis, granulomatosis with polyangiitis, hypertrophic pachymeningitis, pulmonary mass

## Abstract

**Background:**

Granulomatosis with polyangiitis (GPA) is an anti-neutrophil cytoplasmic antibody (ANCA)-associated small-vessel vasculitis classically presenting with upper and lower respiratory tract and renal involvement. Central nervous system (CNS) involvement in GPA is rare and is highly prone to being misdiagnosed as intracranial tumor metastasis or intracranial infection.

**Case report:**

We report a 60-year-old female who presented with recurrent headache and conjunctival injection. She had undergone two surgical procedures for “glaucoma” at an outside hospital without headache relief. Upon admission to our hospital, chest CT revealed multiple bilateral pulmonary nodules, highly suspicious for lung cancer; brain MRI showed dural thickening with subdural effusion; lumbar puncture revealed an intracranial pressure (ICP) of 270 mmH₂O, with negative CSF cultures and no tumor cells. The initial diagnosis was leptomeningeal metastasis from lung cancer. However, percutaneous lung biopsy showed no evidence of tumor or infection, and serology revealed positive c-ANCA/PR3-ANCA, leading to a final diagnosis of GPA with clinicoradiologically supported GPA-related pachymeningitis. After glucocorticoid therapy, the patient's headache and other symptoms resolved rapidly.

**Literature review:**

We reviewed 33 published cases of GPA with cranial involvement and summarized their clinical features. Hypertrophic pachymeningitis (HP) was the most common cranial manifestation (51.5%). Serum ANCA positivity was 87.9% (29/33). Among all 33 cases, c-ANCA/PR3-ANCA was reported in 23 (69.7%); among the 29 ANCA-positive cases, c-ANCA/PR3-ANCA accounted for 79.3% (23/29). After immunosuppressive or immunomodulatory therapy, approximately 90.9% of patients improved or stabilized. These features may serve as clues that should prompt clinicians to consider GPA in the differential diagnosis of pulmonary nodules with pachymeningeal disease and raised intracranial pressure, rather than as established discriminators from lung cancer brain metastasis.

**Conclusion:**

GPA can present with multiple pulmonary masses, headache, and pachymeningitis, closely mimicking leptomeningeal metastasis of lung cancer. When imaging and clinical presentation are atypical, serum ANCA testing and pathological biopsy are key to differential diagnosis. Clinicians should be alert to this “mimic of malignancy” to avoid unnecessary surgery or radiotherapy.

## 1 Introduction

GPA, formerly known as Wegener's granulomatosis, is an ANCA-associated autoimmune disease. Its classic triad involves the upper respiratory tract, lungs, and kidneys, but GPA can affect any organ system (1). Because the manifestations of GPA in specific organs closely resemble primary diseases of those organs (such as infections or tumors) (2), and because of its low incidence, the diagnosis is often challenging, with a mean diagnostic delay of months to years (3).

Notably, when GPA simultaneously involves the lungs and the CNS, its imaging and clinical features can be highly similar to those of lung cancer with intracranial metastasis, leading to frequent misdiagnosis (4). In addition, ocular involvement is not uncommon in GPA but is often mistaken for infectious eye disease or glaucoma (5). Here, we report a case that presented with intractable headache, conjunctival congestion, and bilateral pulmonary masses, ultimately diagnosed as GPA. The classic triad was not clinically apparent at presentation; however, nasopharyngoscopy revealed bilateral pale and edematous nasal mucosa and an erosive lesion at the posterior nasal septum, indicating upper-airway involvement. We also review the literature on GPA with cranial involvement, aiming to improve clinicians' recognition of this “tumor mimic”.

## 2 Case report

A 60-year-old female developed headache (predominant on the right side) without obvious cause in October 2025, accompanied by bilateral conjunctival injection, photophobia, tinnitus, and cough with sputum. After symptomatic treatment at an outside hospital, the respiratory symptoms partially improved. In January 2026, headache and ocular symptoms worsened, and she was diagnosed with “glaucoma” at a local hospital. She underwent two anti-glaucoma surgeries (specific procedures unknown) on January 4 and January 30, 2026; symptoms improved transiently but recurred shortly after, and oral ibuprofen was ineffective. The details of the two previous anti-glaucoma procedures and the original ophthalmological records were not available. True glaucoma, episcleritis, scleritis, uveitis, and orbital disease could not be definitively excluded. During the course, she had no epistaxis, fever, or night sweats, but her body weight decreased by about 9 kg over 2 months. Past history: ureteroscopic nephrolithotomy in 2024; no history of autoimmune disease or hypertension.

On March 5, 2026, she was admitted to our hospital due to persistent severe headache. Chest and abdominal CT showed multiple bilateral pulmonary nodules, suggestive of metastases. Cranial MRI with contrast revealed bilateral dural thickening and enhancement, with right fronto-parieto-occipital subdural effusion; infectious etiology needed to be ruled out (Figures 1a-f). Laboratory tests: WBC 10.17 × 10⁹/L, neutrophils 7.77 × 10⁹/L, hemoglobin 88 g/L, platelets 559 × 10⁹/L; ESR ≥120 mm/h, CRP 80.1 mg/L; serum creatinine and urea were normal, albumin 31.5 g/L; urinalysis showed leukocytes (+) with negative occult blood and negative protein. Urine microscopy for dysmorphic red cells and red-cell casts, quantification of proteinuria (24-h urine protein or urine protein/creatinine ratio), and eGFR were not performed. Renal involvement therefore could not be definitively excluded. The initial clinical diagnosis was lung cancer with brain metastasis, and she was transferred to the oncology department.

**Figure 1:**
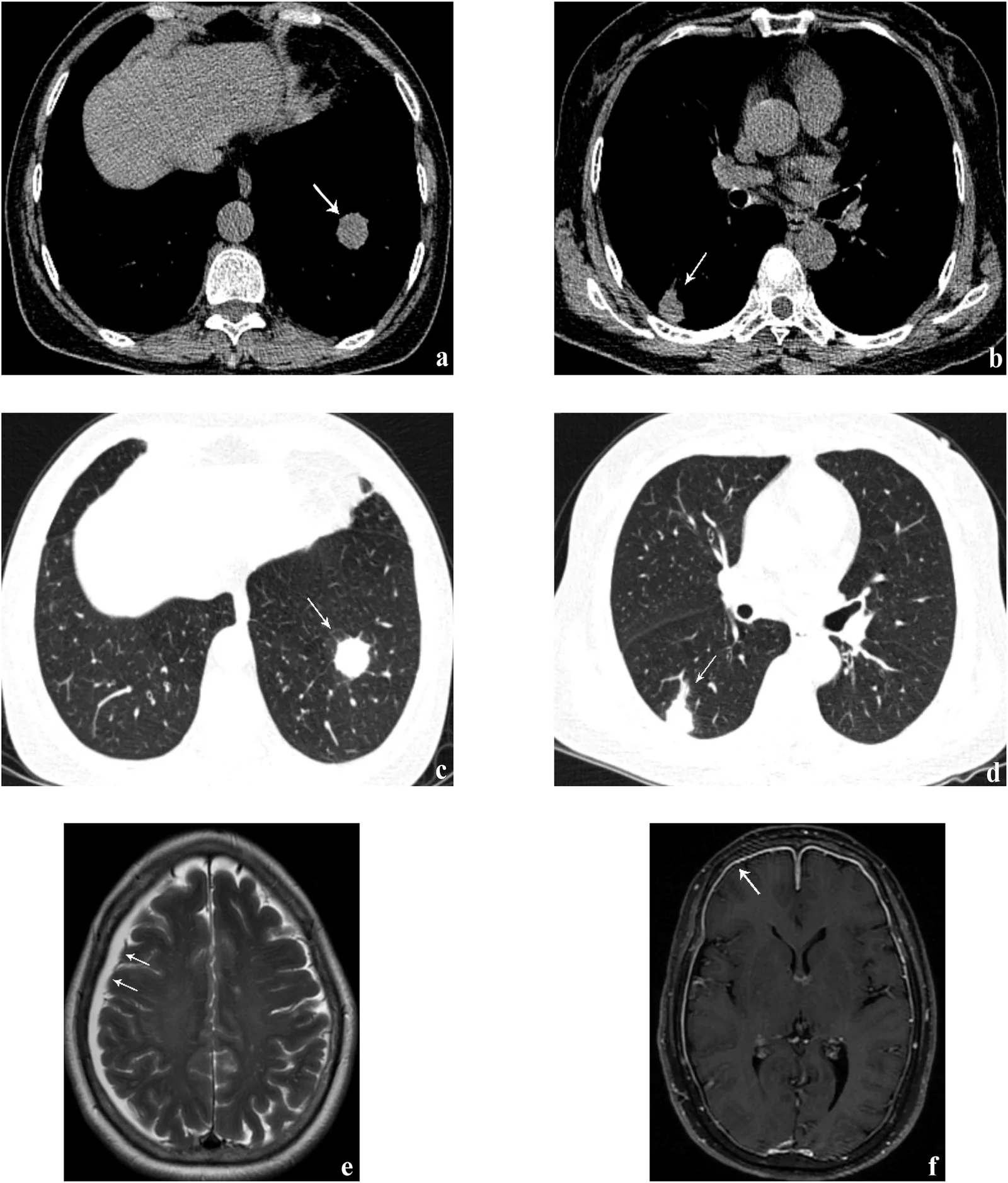

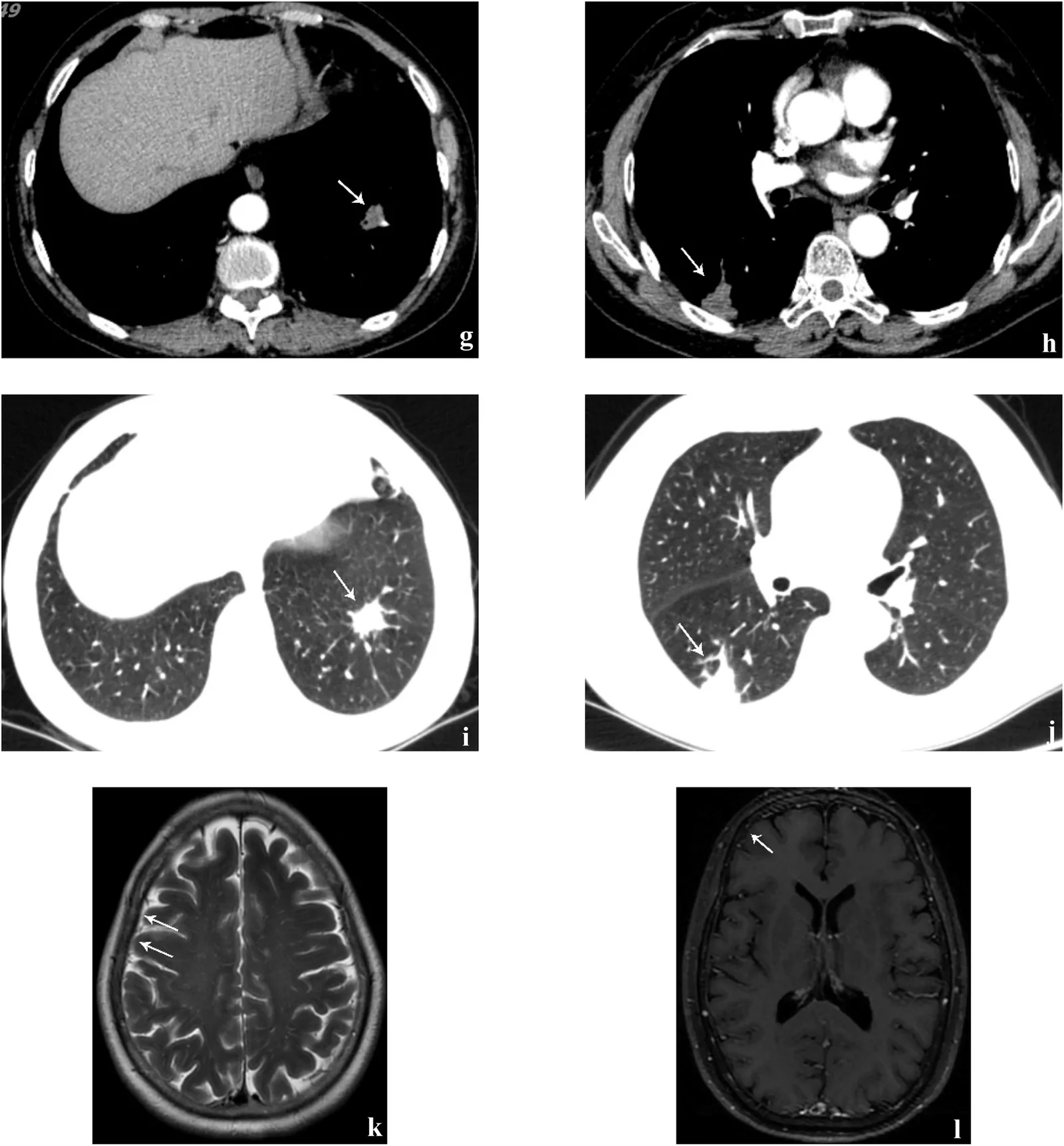
Changes in chest CT and brain MRI before and after treatment: **(a-d)**. Chest CT on 2026-3-5 showing multiple bilateral pulmonary masses with surrounding “spiculation”, resembling lung cancer; **(e,f)**. Contrast-enhanced brain MRI on 2026-3-6 showing diffuse dural thickening and enhancement with right fronto-parieto-occipital subdural effusion; **(g-j)**. Chest CT on 2026-4-14 showing partial regression of pulmonary masses; **(k,l)**. Contrast-enhanced brain MRI on 2026-4-15 showing markedly reduced dural enhancement compared with before.

After transfer, respiratory pathogen tests (influenza virus, Legionella, Mycoplasma, fungal G/GM tests, T-SPOT for tuberculosis) and tumor markers (CEA, CA125, CYFRA21-1, NSE, SCC) were all negative or within normal ranges. Ophthalmological consultation on March 9, 2026 showed best-corrected visual acuity of 0.6 in the right eye and 1.0 in the left eye; intraocular pressure was 17 mmHg in the right eye and 18 mmHg in the left eye. Slit-lamp examination revealed conjunctival hyperemia in both eyes, two peripheral iridotomy openings in the superior iris of the right eye, and a filtering bleb superiorly in the right eye. The cornea was clear, the anterior chamber depth was normal, the pupil was round with a light reflex, and grade II nuclear opacity was present in both eyes. B-scan ultrasonography showed vitreous opacity and a rough retina in both eyes. The ophthalmologist recommended treatment of the underlying systemic disease and stabilization of the patient's general condition. On March 10, 2026, the first lumbar puncture showed an opening pressure of 270 mmH₂O, clear fluid, white blood cell count 1 × 10⁶/L, red blood cell count 10 × 10⁶/L, and a negative Pandy test; CSF protein, glucose, and chloride were normal. Bacterial and fungal cultures were negative, and no tumor cells were detected. Mannitol dehydration and dexamethasone (5 mg/d × 3 days) provided slight headache relief. On March 11, 2026, CT-guided percutaneous lung biopsy was performed; pathology reported necrotizing granulomatous inflammation with inflammatory cell infiltration in vessel walls; special stains (PAS, PAM, acid-fast, and auramine O fluorescence) were negative for fungi and acid-fast bacilli, TB PCR was negative, and no tumor cells were seen. Given the vasculitic pathology, serology revealed positive c-ANCA and PR3-ANCA >200 RU/mL (strongly positive), with negative MPO-ANCA. Electronic nasopharyngoscopy showed bilateral pale and edematous nasal mucosa and an erosive lesion at the posterior nasal septum. Based on pulmonary histology and strongly positive PR3-ANCA, systemic GPA was confirmed. Because the dural lesion was not biopsied, cranial involvement was diagnosed as clinicoradiologically supported GPA-related pachymeningitis. On March 22, 2026, a second lumbar puncture showed clear fluid with an opening pressure of 170 mmH₂O (decreased after mannitol dehydration); bacterial and fungal cultures were negative, and no Cryptococcus neoformans was detected. Repeated cytology and flow cytometry were not performed.

On March 24, 2026, methylprednisolone (80 mg/d intravenously) was started; the next day, headache was essentially relieved and conjunctival congestion markedly subsided. After 3 days, she was switched to oral prednisone (60 mg/d, tapered weekly). At discharge on March 28, 2026, she was headache-free and in good mental status. On April 14, 2026, follow-up chest CT showed that the multiple patchy and nodular opacities had significantly decreased; brain MRI showed marked improvement in dural thickening and enhancement, and the previous right subdural effusion had largely resolved (Figures 1g-l) (Figure 2).

**Figure 2:**
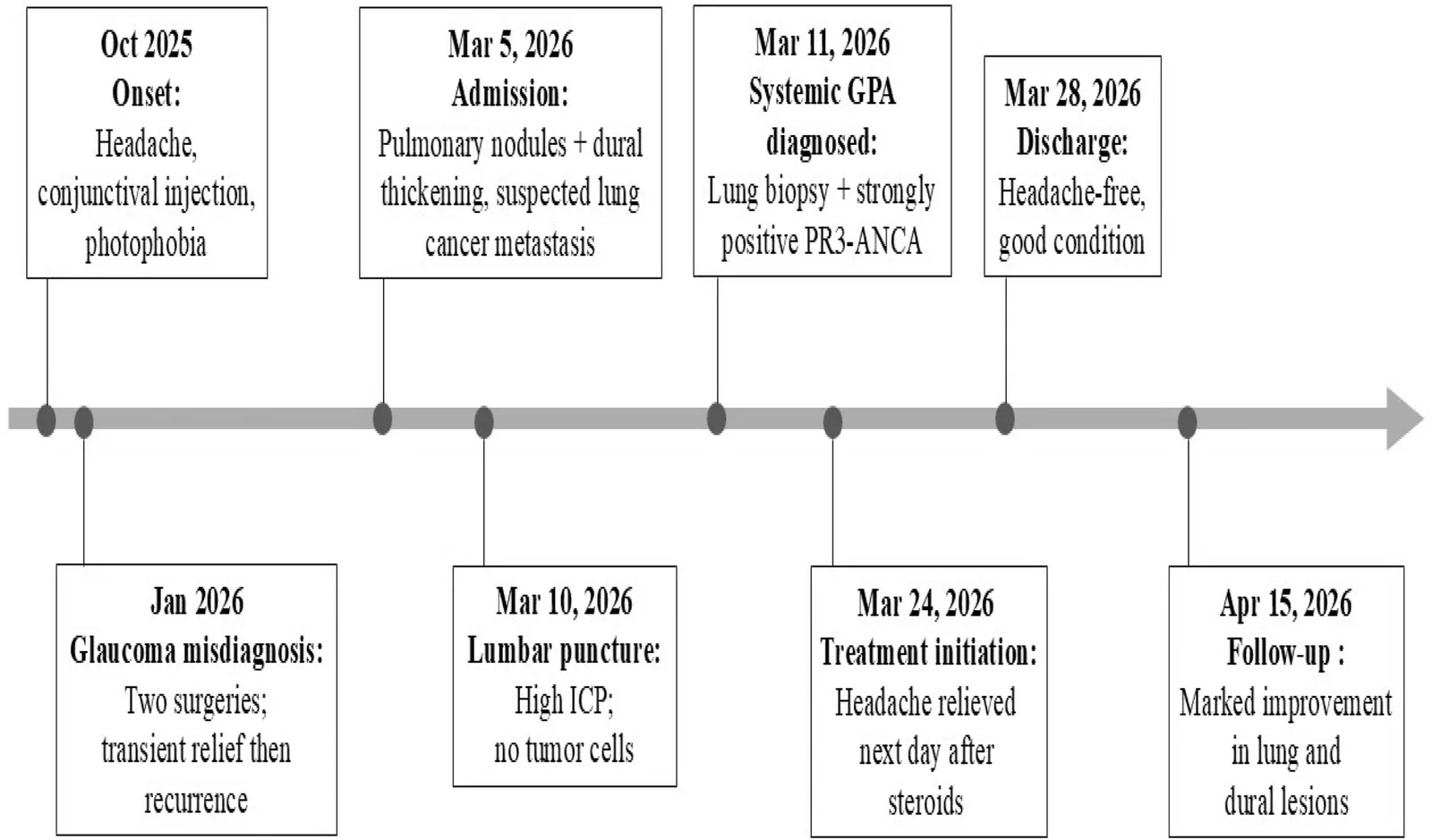
Structured clinical timeline. The patient developed headache, conjunctival injection, photophobia, tinnitus, and cough in October 2025, and was misdiagnosed with glaucoma in January 2026. On admission (March 5, 2026), pulmonary nodules and dural thickening mimicked lung cancer with meningeal metastasis. Lumbar puncture (March 10, 2026) showed elevated opening pressure, normal protein/glucose, and no tumor cells. Lung biopsy (March 11, 2026) plus strongly positive PR3-ANCA confirmed systemic GPA; cranial involvement was defined as clinicoradiologically supported GPA-related pachymeningitis because the dural lesion was not biopsied. Methylprednisolone was started on March 24, 2026, with headache relief the next day; she was discharged on March 28, 2026. Follow-up on April 14–15, 2026 showed marked improvement in pulmonary and dural lesions. Follow-up was approximately 1 month; long-term relapse and treatment durability remain unknown.

## 3 Discussion

### 3.1 Diagnostic challenges and clinical presentation

The diagnostic process in this case was extremely challenging, going through two misdiagnostic stages—from “glaucoma” to “lung cancer brain metastasis"—before finally being diagnosed as GPA. The diagnostic difficulties were threefold: first, presentation with headache and ocular symptoms (conjunctival injection) mimicked glaucoma, making unnecessary glaucoma surgery likely; second, bilateral pulmonary masses combined with intracranial hypertension and dural thickening were readily interpreted as classic signs of lung cancer leptomeningeal metastasis; third, although the classic triad was not clinically apparent at presentation, upper-airway findings on nasopharyngoscopy and pulmonary involvement were present, demonstrating strong clinical heterogeneity. This case suggests that when a patient presents with multisystem involvement that does not respond to general treatment or anti-infective/anti-tumor therapy, GPA should be included in the differential diagnosis even in the absence of typical symptoms. In this case, systemic GPA was confirmed by pulmonary histology and strongly positive PR3-ANCA, whereas the cranial involvement was defined as clinicoradiologically supported GPA-related pachymeningitis because the dural lesion was not biopsied.

### 3.2 Literature review of GPA with cranial involvement

We performed a narrative literature review of published cases of GPA with cranial involvement. PubMed was searched up to December 2025 using the terms “granulomatosis with polyangiitis”, “Wegener's granulomatosis”, “central nervous system”, “cranial”, “pachymeningitis”, “hypophysitis”, and “cerebral vasculitis”. Because a formal systematic review was not intended, no PRISMA flow diagram or duplicate screening process was applied; this is acknowledged as a limitation. We included published cases of GPA with definite cranial/CNS lesions (pachymeningitis, hypophysitis, cerebral vasculitis, etc.). The 2022 ACR/EULAR criteria for GPA are classification rather than diagnostic criteria (6). Because many included cases were published before 2022 and the review focused on atypical CNS disease that may mimic other disorders, we accepted the diagnosis as stated in each original report and recorded the diagnostic basis where available. A total of 33 evaluable cases were identified (Table 1) (7–36). The results were as follows: median age 42 years (range 9-68), male-to-female ratio approximately 1:1. Serologically, overall ANCA positivity was 87.9% (29/33). Among all 33 cases, c-ANCA/PR3-ANCA was reported in 23 (69.7%), MPO-ANCA in 3 (9.1%), p-ANCA without a specified subtype in 2 (6.1%), ANCA positivity without a specified subtype in 1 (3.0%), and ANCA negativity in 4 (12.1%), indicating that ANCA negativity does not exclude cranial GPA. Imaging findings most commonly showed HP (51.5%, 17/33), typically involving the falx cerebri, tentorium, and skull base; followed by cerebrovascular events (30.3%, including ischemic stroke and venous thrombosis) and hydrocephalus (15.2%). Regarding treatment, glucocorticoids were the most basic therapy (93.9%), with cyclophosphamide (63.6%), rituximab (33.3%), and azathioprine (24.2%) as common immunosuppressants. Prognostically, about 90.9% (30/33) of patients achieved stable or improved clinical status, but some had residual visual or hearing deficits; two patients died (status epilepticus and septic shock). Our case's clinical presentation (clinicoradiologically supported GPA-related pachymeningitis with subdural effusion and high ICP) was consistent with the most common cranial involvement pattern reported in the literature. Because the included cases were selected for GPA and no comparator group was available, these features cannot establish how well they discriminate GPA from lung cancer meningeal metastasis.

**Table 1:** Clinical characteristics of reported cases of GPA with cranial involvement.

No.	Case	Age/Sex	Neurological symptoms	Imaging findings	ANCA results	Diagnostic basis	Treatment	Outcome
1	Lučić, 2015 (7)	41/M	Headache, diplopia	Multiple cortical infarcts	PR3-ANCA+	Biopsy+serology	CYC + MP→AZA	No recurrence at 24-month follow-up
2	Seror, 2006 (8)	54/M	Headache	Dural thickening and enhancement of tentorium and falx cerebri, hydrocephalus of third ventricle	p-ANCA+	Clinical-radiological+serology	CYC→AZA + GC	Headache resolved,MRI partially improved
3	Seror, 2006 (8)	42/M	Headache, seizures, behavioral changes, hemiparesis	Diffuse dural thickening and enhancement, hydrocephalus, cerebral venous thrombosis	c-ANCA+	Biopsy + serology	CYC + GC→biologic agent + shunting	Died of status epilepticus
4	Seror, 2006 (8)	35/M	Abducens nerve palsy, cerebellar nystagmus, coma	Cerebellar hematoma, ventricular dilatation	ANCA-	Biopsy	Surgery, drainage, CYC + GC→AZA	Symptoms resolved, residual imaging abnormalities
5	Bonek, 2023 (9)	68/M	Cranial nerve palsies (VI-XI), cerebellar symptoms, headache	Skull base mass, cerebellar sinus thrombosis	c-ANCA+	Biopsy + serology	CYC + GC→RTX + MMF	Clinical remission, mass regression
6	Tishler, 1993 (10)	41/M	Headache, blurred vision, seizures	Nodular meningeal enhancement	c-ANCA+	Biopsy + serology	CYC	Symptoms improved, partial resolution of meningeal enhancement at 16 months
7	Yasuhara, 2002 (11)	28/M	Headache, fever, nuchal rigidity	Linear meningeal thickening and enhancement	c-ANCA+	Biopsy + serology	MP→CYC + GC	Headache resolved, leptomeningeal thickening regressed
8	Azin, 2023 (12)	12/F	Headache, blurred vision, loss of consciousness, papilledema	PRES (diffuse T2 hyperintensity at grey-white matter junction with hemorrhage)	c-ANCA+	Biopsy + serology	MP + RTX→GC + MMF	Lesions resolved, residual chronic kidney disease
9	Yildirim, 2008 (13)	65/M	Vertigo, gait disturbance, meningeal signs	Cochlear and labyrinthine enhancement, frontal leptomeningeal FLAIR hyperintensity	ANCA-	Clinical-radiological	Intratympanic PSL, oral GC and MTX	Mild hearing improvement, reduction of labyrinthine enhancement and frontal leptomeningeal hyperintensity
10	Enabi, 2024 (14)	42/F	Headache, abducens nerve palsy	Thickening and enhancement of falx and tentorium, dural enhancement	ANCA+	Biopsy + serology	RTX + MP→Pred + MP	Symptoms improved after 3 days
11	Bernat, 2016 (15)	35/F	Headache, anosmia, diplopia	Mass in olfactory groove and right cribriform plate, frontal dural thickening and enhancement	MPO-ANCA+	Biopsy + serology	Surgical resection→GC + AZA	MRI normal at 1 year post-op
12	Yuan, 2023 (16)	32/F	Dizziness, headache	Fourth ventricle mass	ANCA+, PR3-ANCA+	Biopsy + serology	CYC + MP→RTX + GC + surgery	Pulmonary lesions/skin ulcers completely resolved, brain mass progressed
13	Esposito, 2017 (17)	37/F	Headache, polydipsia, polyuria, amenorrhea, fatigue	Sellar cystic mass	c-ANCA+,PR3-ANCA+	Biopsy + serology	PSL + MTX→CYC	Sellar mass shrank, lifelong hormone replacement
14	Peters, 2018 (18)	32/F	Multiple cranial nerve palsies, central diabetes insipidus, amenorrhea	Extensive inflammatory infiltration of skull base, internal carotid artery occlusion, pituitary stalk thickening and enhancement	c-ANCA+, initial PR3-ANCA + then negative after relapse	Biopsy + serology	MTX + PSL→RTX + GC + CYC→AZA + PSL	Cranial nerve function improved, stable intracranial disease for 20 months
15	de Jesus, 2026 (19)	49/F	Bilateral vision loss, right RAPD+, bilateral hearing loss	HP, optic canal enhancement	ANCA-	Biopsy	CYC→AZA	Vision not recovered, disease stable
16	Kato, 2018 (20)	50/F	Headache, nausea/vomiting, impaired consciousness	HP, SMICH	MPO-ANCA + initially,−at relapse	Biopsy + serology	Initial: PSL, CYC→MTX; at SMICH: MP→PSL	Residual mild higher cerebral dysfunction
17	Tarte, 2020 (21)	47/M	Dysphagia, hoarseness, absent gag reflex	Acute left medullary infarct, acute left centrum semiovale lacunar infarct	c-ANCA+,PR3-ANCA+	Serology	RTX + GC + AZA	Slow improvement in swallowing and hoarseness
18	Nakazawa, 2017 (22)	37/M	Headache, cranial nerves IX–XII palsy	Right skull base infiltrative lesion encasing carotid sheath	PR3-ANCA+	Clinical-radiological + serology	CYC + GC→RTX + GC	Only hoarseness remained at 1 year
19	Kuribayashi, 2019 (23)	58/M	Left facial paresthesia, headache	Left frontal edema, frontotemporal dural thickening and enhancement, superior sagittal sinus stenosis	PR3-ANCA+	Clinical-radiological + serology	MP→Pred + CYC	Symptoms resolved and imaging improved in about 30 days
20	Li, 2024 (24)	33/F	Headache, nuchal rigidity, nausea/vomiting	Fourth ventricle mass with obstructive hydrocephalus	c-ANCA+, PR3-ANCA +	Biopsy + serology	MP + CYC→Pred + CYC→RTX + CYC→surgery→intrathecal MTX/DXM + MP→Pred + MMF + RTX	Headache resolved, fourth ventricle mass significantly reduced
21	Alameri, 2024 (25)	55/F	Blurred vision, bitemporal hemianopia, AVPD	Sellar mass compressing optic chiasm	PR3-ANCA+	Clinical-radiological + serology	PSL→RTX + CYC→RTX maintenance	Vision improved, pituitary lesion shrank
22	Alameri, 2024 (25)	38/F	Headache, left visual field defect, AVPD	Sellar cystic mass, pituitary stalk thickening, optic chiasm displacement, hypothalamic enhancement	PR3-ANCA+	Clinical-radiological + serology	HC→PSL→RTX maintenance	Cyst resolved, pituitary stalk normalized
23	Lankapothu, 2024 (26)	55/F	Hoarseness, dysphagia,dysarthria, tongue deviation to right, absent gag reflex (IX/X/XII palsies)	Middle and posterior fossa dural thickening and enhancement, bilateral IX/X/XII cranial nerve thickening and enhancement	c-ANCA+, PR3-ANCA+	Clinical-radiological + serology	MP→GC + MMF→relapse added CYC	Improved swallowing, tongue movement and saliva pooling, but persistent dysarthria
24	Arnaoutoglou, 2018 (27)	54/M	Headache, hoarseness, left and lower visual field diplopia	Extensive dural thickening	c-ANCA+, p-ANCA-	Biopsy + serology	GC→GC + CYC	MRI normal at 2 years
25	Lu, 2016 (28)	9/F	Lower limb weakness, urinary incontinence, vision loss	Frontoparietal basal ganglia lesions, diffuse meningeal enhancement, sphenoid sinusitis	MPO-ANCA+	Clinical-radiological + serology	MP + IVIG→MP + MMF	No relapse at 1.5 years, vision recovered to 1.0
26	Von Scheven, 1998 (29)	13/F	Seizures, confusion, pyramidal signs	Multiple white matter T2 hyperintensities	c-ANCA+	Biopsy + serology	Pred + CYC→MTX	No seizures at 1.5 years
27	Jouret, 2008 (30)	52/M	Syncope, confusion, dysarthria, photophobia, hyperacusis, headache	Right frontal pseudotumoral mass with extensive vasogenic edema	c-ANCA+	Biopsy	MP + CYC	Symptoms resolved and intracranial mass shrank at 3 months
28	Parikh, 2025 (31)	30/F	Headache, seizures	Vasogenic edema, PRES	PR3-ANCA+	Biopsy + serology	GC + RTX	Improved
29	Dörr, 2008 (32)	46/M	Headache, left papilledema	Left temporoparietal dural thickening with enhancement	PR3-ANCA+	Biopsy + serology	MP→CYC + PSL→AZA + PSL	Headache immediately relieved, partial resolution of dural enhancement, clinically stable at 12 months
30	Huaringa-Marcelo, 2020 (33)	60/M	Headache, vomiting, papilledema, III/VI cranial nerve palsies	Convexity and tentorial dural thickening and enhancement	PR3-ANCA-, MPO-ANCA-	Biopsy	Pred	Clinically improved and discharged (died of septic shock 1 week after discharge)
31	Wade, 2026 (34)	19/F	Headache, right hearing loss, blurred vision, right abducens palsy (VI)	Basilar dural thickening and enhancement	c-ANCA+, PR3-ANCA+	Biopsy + serology	DXM (further treatment not specified)	Transient improvement after dexamethasone
32	Pensato, 2020 (35)	55/M	Headache, papilledema	Diffuse dural thickening and enhancement	c-ANCA+, p-ANCA+	Biopsy + serology	MP→PSL	Headache resolved, dural thickening and enhancement reduced
33	Barbieri, 2018 (36)	61/F	Headache, hearing loss	Posterior fossa dural thickening and enhancement, cerebellar tonsillar herniation, acute non-communicating hydrocephalus of third ventricle	p-ANCA+	Biopsy + serology	MP + plasma exchange→relapse: ETV→MP maintenance	Symptoms immediately improved after ETV, symptoms resolved at 1-year follow-up, persistent posterior fossa pachymeningitis

CYC, cyclophosphamide; AZA, azathioprine; GC, glucocorticoid; MP, methylprednisolone; RTX, rituximab; MMF, mycophenolate mofetil; PSL, prednisolone; MTX, methotrexate; Pred, prednisone; PRES, posterior reversible encephalopathy syndrome; RAPD, relative afferent pupillary defect; SMICH, simultaneous multiple intracerebral hemorrhages; AVPD, arginine vasopressin deficiency; HC, hydrocortisone; IVIG, intravenous immunoglobulin; DXM, dexamethasone; ETV, endoscopic third ventriculostomy. Diagnostic basis was recorded as reported in the original articles: “Biopsy” indicates histopathological confirmation of GPA; “Serology” indicates ANCA positivity used as supportive evidence; “Clinical-radiological” indicates diagnosis based on compatible clinical and imaging findings without biopsy confirmation.

### 3.3 Mechanism of intracranial hypertension and subdural effusion

In our patient with clinicoradiologically supported GPA-related pachymeningitis, significant intracranial hypertension (270 mmH₂O) and right fronto-parieto-occipital subdural effusion were observed, the mechanisms of which warrant further exploration. Several related pathological processes may be involved: ① CSF absorption impairment: HP can involve the arachnoid granulations, hindering CSF drainage into the superior sagittal sinus, leading to communicating hydrocephalus and secondary subdural effusion – this is the most likely mechanism (37). ② Venous sinus outflow obstruction: dural inflammation can extend to the venous sinus wall, inducing inflammatory stenosis or thrombosis, further exacerbating intracranial hypertension (38). ③ Increased local inflammatory exudation: active pachymeningitis produces inflammatory exudates that accumulate in the subdural space (39). In our case, normal CSF protein, a decline in opening pressure from 270 mmH₂O to 170 mmH₂O after mannitol dehydration, and rapid absorption of the effusion support inflammatory exudation rather than CSF leakage. ④ Granulomatous mass effect: in rare cases, granulomas can directly obstruct CSF pathways, causing hydrocephalus and elevated pressure (24). However, no definite obstruction was seen on imaging in our case. This case suggests that for GPA-related HP, subdural effusion is not an absolute indication for surgical drainage; effective immunosuppressive therapy can reverse it.

### 3.4 Differential diagnosis: GPA vs. lung cancer meningeal metastasis

In our case, the initial misdiagnosis as lung cancer leptomeningeal metastasis was highly likely, but key differentiating points exist across multiple dimensions (Table 2). At the clinical decision-making level, the following two points are decisive: First, serum ANCA testing provides a crucial clue. In our case, before pathology pointed to vasculitis, the strong positivity for c-ANCA/PR3-ANCA strongly suggested GPA and guided timely glucocorticoid therapy. Second, pathological biopsy is the gold standard, but it is important to note that lung biopsy in GPA may only show necrotizing granulomas and vasculitis; oncologists and pathologists must be aware of this, avoiding misinterpretation as “atypical hyperplasia of unknown significance”. Furthermore, treatment response should be interpreted as supportive rather than diagnostic. Corticosteroids can also produce rapid symptomatic improvement in intracranial malignancy by reducing peritumoral edema; indeed, this patient's headache improved slightly with dexamethasone while leptomeningeal metastasis was still the working diagnosis. Therefore, the rapid response to glucocorticoids cannot by itself distinguish GPA from lung cancer meningeal metastasis.

**Table 2:** Differential diagnosis between GPA with cranial involvement and lung cancer meningeal metastasis.

Feature	GPA with cranial involvement	Lung cancer meningeal metastasis
Pulmonary nodule characteristics	Bilateral, multiple, may be cavitary or migratory	Primary mass present; metastatic lesions are solid with few cavities
Site of meningeal involvement	Dura mater (diffuse or focal thickening and enhancement)	Leptomeninges/pia-arachnoid (linear or nodular enhancement)
Cerebrospinal fluid findings	Pressure may be elevated; cytology (−); protein normal or mildly elevated	Pressure elevated; cytology (+); protein markedly elevated, glucose decreased
Serum ANCA	Mostly positive (c-ANCA/PR3-ANCA elevated)	Negative
Pathology	Necrotizing granuloma + vasculitis; no tumor cells	Atypical cell nests (tumor cells) present

## 4 Therapeutic response and implications

For GPA patients with CNS involvement, especially those with HP and elevated ICP, immunosuppressive therapy should be initiated as early as possible (40). In this patient, moderate-dose intravenous methylprednisolone (80 mg/d) followed by oral prednisone produced rapid clinical and radiological improvement. However, this observation should not be generalized: CNS involvement in ANCA-associated vasculitis may be organ-threatening, and current recommendations, including the 2021 ACR/Vasculitis Foundation guideline, support combination induction therapy with glucocorticoids plus rituximab or cyclophosphamide for severe organ-threatening disease (41). The regimen used here should therefore be regarded as a case-specific decision rather than a general recommendation. Close follow-up is required, and cyclophosphamide or rituximab should be added promptly if relapse occurs during steroid tapering.

For symptomatic management of intracranial hypertension, short-term mannitol dehydration may be used, but definitive treatment relies on immunosuppression. If drug-refractory hydrocephalus or massive subdural effusion with neurological deterioration develops, ventricular or subdural drainage may be considered (37). In our patient, mild-to-moderate subdural effusion regressed after systemic immunosuppressive treatment, and no surgical drainage was required. Whether this can obviate surgery in general cannot be established from a single case.

## 5 Limitations

This study has several limitations: First, as a single case report, it cannot represent the full spectrum of GPA with CNS involvement, and the generalizability is limited. Second, although clinical and serological evidence strongly supported GPA, the meningeal lesion was not biopsied for direct pathological confirmation. Third, although two lumbar punctures were performed, repeated CSF cytology and flow cytometry were not performed; therefore, leptomeningeal metastasis could not be fully excluded by CSF analysis alone. Fourth, the follow-up duration was only about 1 month; long-term relapse rate, treatment durability, and late outcomes cannot be determined, and the observed improvement should be regarded as a short-term response rather than sustained remission. Fifth, the literature review included mostly retrospective case reports, which may be subject to publication bias. In addition, the literature review was not conducted with full systematic-review methodology (single database, no duplicate screening, and no PRISMA flow diagram), which may introduce selection and reporting bias. Future large-scale prospective studies are needed to define the optimal treatment strategy for GPA with CNS involvement.

## 6 Conclusion

GPA can present with headache, intracranial hypertension, pachymeningitis, and multiple bilateral pulmonary nodules, closely mimicking lung cancer with meningeal metastasis on imaging. When encountering pulmonary nodules combined with dural thickening, subdural effusion, or elevated ICP, and CSF and pathology show no tumor cells, serum ANCA (especially c-ANCA/PR3-ANCA) should be tested promptly, and GPA should be highly suspected. Clinicians should be aware of this “tumor mimic”, because considering GPA early may potentially avoid unnecessary surgery or chemoradiotherapy and may potentially improve outcomes. However, this potential benefit cannot be established from a single case and a small case-report literature review, and prospective studies are needed.

## Data Availability

The original contributions presented in the study are included in the article/Supplementary Material, further inquiries can be directed to the corresponding authors.
